# Supplementary material for: ATR is required to complete meiotic recombination in mice
Source: Nat Commun. 2018 Jul 5;9:2622. doi: 10.1038/s41467-018-04851-z (PMC6033890; doi:10.1038/s41467-018-04851-z)
Supplement: Supplementary file 1 — Supplementary Information [file 41467_2018_4851_MOESM1_ESM.pdf]

ATR is required to complete meiotic recombination in mice

Pacheco et al.

Supplementary Information

Supplementary Figure 1

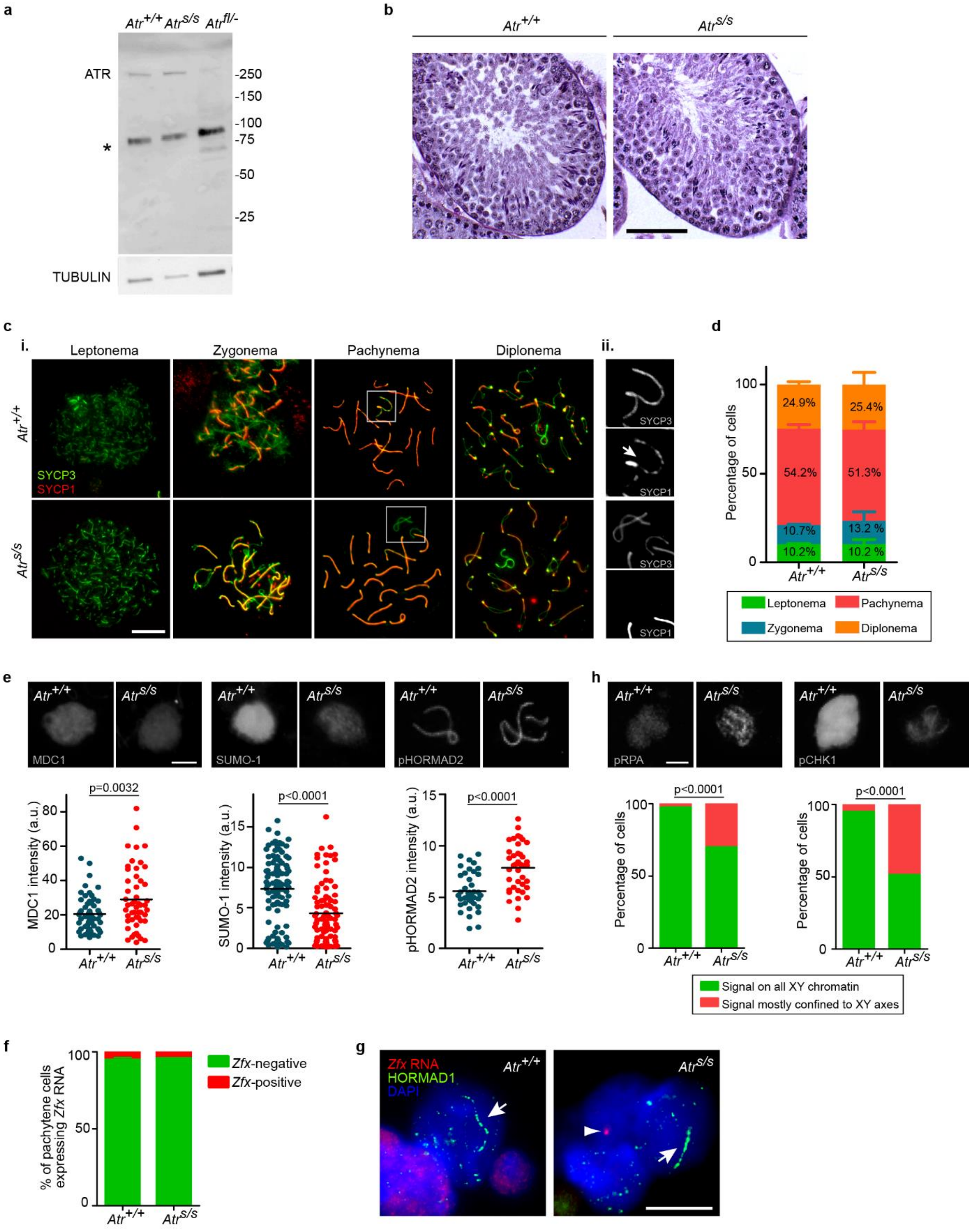

Su

Supplementary figure 1.

***Atr$^{S/S}$* testes show abnormal sex-body formation.**

(**a**) Western blots of whole testis extracts from *Atr$^{+/+}$*, *Atr$^{S/S}$* and *Atr$^{fl/-}$* adult mice. Asterisk indicates non-specific signal. As previously described[14], adult Seckel mouse testis shows wild-type levels of ATR protein. Note that the truncated ATR form predicted from skipping of exon 9 (expected molecular mass of ~70 kDa) was not detected in the Seckel sample. Testis extracts from a conditional *Atr* mutant (*Atr$^{fl/-}$ Ngn3-Cre* from Widger et al[52]) were used to test antibody specificity. (**b**) Seminiferous tubule cross-sections from *Atr$^{+/+}$* and *Atr$^{S/S}$* stained with PAS-hematoxylin contain multiple spermatogenic stages, from spermatogonial stem cells at the base of the tubule to elongated spermatids at the lumen. (**ci**) Representative images from *Atr$^{+/+}$* and *Atr$^{S/S}$* spermatocytes immunostained for SYCP3 and SYCP1. Scale bar represents 10 µm. *Atr$^{S/S}$* spermatocytes complete autosomal synapsis, however note the presence of unsynapsed X and Y chromosomes in *Atr$^{S/S}$* spermatocytes at pachynema (white box). (**cii**) Magnified images of sex chromosomes at pachynema (white boxes). In control spermatocytes, sex-chromosome synapsis is demonstrated by the presence of SYCP1 in the PAR (arrow). *Atr$^{S/S}$* spermatocytes exhibit unaligned sex chromosomes presenting only SYCP3 staining. (**d**) Percentages of cells at different prophase stages from the indicated genotypes. Columns and lines indicate the mean and SD. Data obtained from the analysis of 2 mice per each genotype. Similar proportions of spermatocytes were found at each stage of meiotic prophase in *Atr$^{+/+}$* and *Atr$^{S/S}$* (N=402 and N=370 respectively, p=0.62, G test). (**e**) Quantification of the intensity of MDC1, SUMO-1, and pHORMAD2 staining on the sex body in arbitrary units (a.u.). Horizontal black lines denote the means. Images show representative sex bodies from *Atr$^{+/+}$* and *Atr$^{S/S}$* pachytene spermatocytes immunostained for MDC1, SUMO-1, or pHORMAD2. Images were captured with the same exposure time. Scale bar represents 2 µm and applies to all panels. (**f**) Percentage of cells exhibiting *Zfx* RNA FISH signals in *Atr$^{+/+}$* (5%, N=100; from 2 mice) and *Atr$^{S/S}$* (4%, N=50, 1 mouse; p=1.0, Fisher's exact test). (**g**) Representative early-pachytene spermatocytes stained for HORMAD1, which is confined to sex chromosomes (arrows) at early pachynema, overlayed with RNA FISH for the X-linked gene *Zfx* and DAPI. Note that *Zfx* was not expressed in the pachytene spermatocytes shown, indicative of proper MSCI in both genotypes. (One HORMAD1-negative cell expressing the X-linked gene is shown (arrowhead)). (**h**) Percentage of sex bodies displaying pRPA or pCHK1 signal mostly located around the axis of the sex chromosomes or spread over the X and Y chromatin. Images show representative sex bodies from *Atr$^{+/+}$* and *Atr$^{S/S}$* pachytene spermatocytes immunostained against pRPA or pCHK1. Images were captured with the same exposure time. Scale bar represents 2 µm and applies to all panels.

Supplementary Figure 2

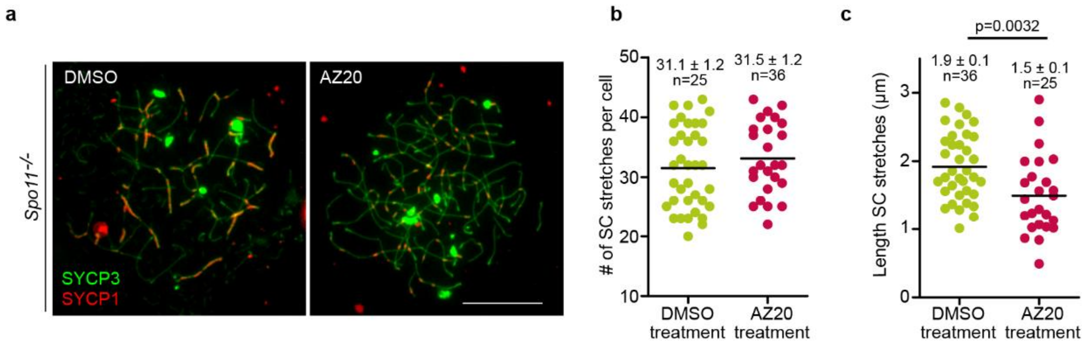

Supplementary figure 2.

***In vivo* inhibition of ATR inhibits SC elongation in *Spo11*$^{-/-}$ spermatocytes.**

(**a**) Representative images from *Spo11*$^{-/-}$ spermatocytes treated with 5 μM AZ20 or the equivalent volume of DMSO for 7 days. Scale bar represents 10 μm. (**b–c**) Quantification of the number (**b**) and the length (**c**) of SC stretches. Horizontal lines denote the means. P values are from t tests.

Supplementary Figure 3

ATR WB

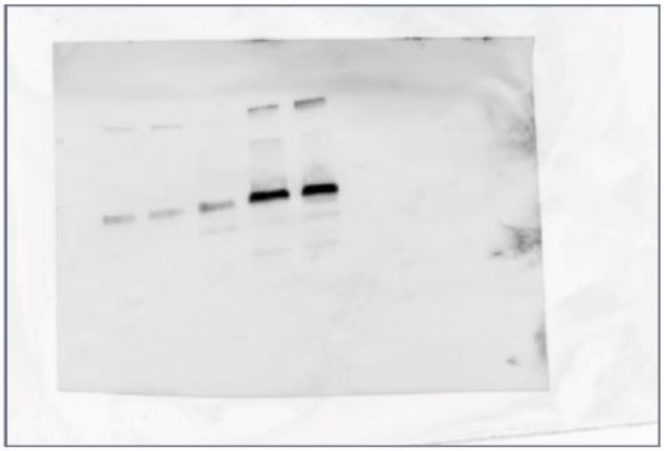

TUBULIN WB

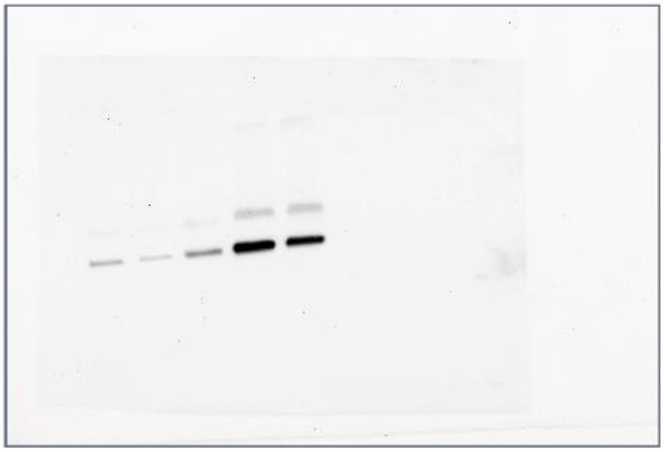

Supplementary figure 3.
Unmodified and uncropped western blots used in Supplementary Figure 1.

Supplementary table 1. Average number of recombination foci present in spermatocytes at different stages of meiotic prophase from the indicated genotypes.

		Early leptonema	Late leptonema	Early zygonema	Late zygonema	Pachynema	Diplonema
RAD51	*Atr*$^{+/+}$	187.6 ± 66.4, N=22	165.8 ± 48.5, N=12	162.0 ± 42.6, N=15	78.5 ± 82.8, N=38	21.0 ± 14.8, N=61	13.1 ± 11.4, N=15
	Atr$^{S/S}$	120.8 ± 62.0, N=12	151.4 ± 47.7, N=16	144.5 ± 39.2, N=53	82.8 ± 27.24, N=79	29.9 ± 16.2, N=104	11.9 ± 8.4, N=29
DMC1	*Atr*$^{+/+}$	181.0 ± 90.1, N=12	163.1 ± 40.2, N=9	118.5 ± 31.6, N=32	65.81 ± 22.6, N=58	19.3 ± 16.0, N=77	2.07 ± 2.7, N=28
	Atr$^{S/S}$	118.3 ± 38.6, N=22	95.2 ± 27.7, N=18	100.3 ± 33.5, N=27	57.73 ± 17.5, N=56	31.2 ± 17.2, N=80	3.2 ± 3.2, N=13
RPA	*Atr*$^{+/+}$	156.8 ± 45.4, N=15	152.5 ± 28.16, N=12	178.2 ± 28.6, N=18	121.4 ± 29.1, N=42	62.2 ± 36.2, N=58	4.9 ± 4.8, N=18
	Atr$^{S/S}$	193.0 ± 52.5, N=17	156.8 ± 22.9, N=19	132.9 ± 33.6, N=15	113.1 ± 15.6, N=41	56.8 ± 31.7, N=79	3.2 ± 3.0, N=24
		**Late zygonema**	**Early pachynema**	**Late pachynema**			
RNF212	*Atr*$^{+/+}$	97 ± 20.3, N=20	78.1 ± 12.6, N=18	39 ± 15.9, N=21			
	Atr$^{S/S}$	110.2 ± 14.5, N=11	87.4 ± 17, N=25	60.8 ± 12.5, N=23			

Mean, standard deviation and total number of cells counted (N) are shown.

Supplementary table 2. Average number of recombination foci in spermatocytes from mice treated with AZ20 or the control vehicle DMSO at the indicated stages of prophase.

		Early leptonema	Late leptonema	Early zygonema	Late zygonema	Pachynema
RAD51	DMSO treatment	173.8 ± 47.3, N=16	184.6 ± 36.0, N=26	153.3 ± 32.31, N=31	91.41 ± 27.5, N=41	7.3 ± 6.0, N=50
	AZ20 treatment	129.1 ± 49.0, N=19	131.2 ± 51.9, N=35	131.5 ± 33.7, N=64	86.2 ± 44.4, N=40	19.3 ± 9.0, N=44
RPA	DMSO treatment	199.1 ± 30.3, N=21	177.2 ± 35.5, N=24	182.8 ± 52.5, N=34	131.5 ± 35.5, N=31	
	AZ20 treatment	238.2 ± 56.6, N=20	155.6 ± 33.5, N=20	127.6 ± 31.1, N=33	97.74 ± 26.5, N=38	
		**Late zygonema**	**Early pachynema**	**Late pachynema**		
RNF212	DMSO treatment	99.3 ± 15.7, N=22	63.5 ± 9.5, N=40	35.6 ± 16.6, N=14		
	AZ20 treatment	95.4 ± 16.5, N=23	71.1 ± 17.4, N=19	51.9 ± 16.4, N=15		

Mean, standard deviation and total number of cells counted (N) are shown.

Supplementary table 3. Average percentage of spermatocytes and proportions of cells in meiotic prophase stages in cultured samples.

		% Spermatocytes	% Leptonema	% Zygonema	% Pachynema	N
Untreated	D0	0.70 ± 1.1, N=709	100.0 ± 0.0	–	–	3
	D7	11.5 ± 5.4, N=1032	76.1 ± 10.5	23.3 ± 9.7	0.65 ± 1.0	324
	D14	15.1 ± 2.9, N=1214	20.0 ± 3.8	54.5 ± 15.3	25.6 ± 12.7	490
AZ20	DMSO1	14.3 ± 5.6, N=825	24.6 ± 5.3	45.1 ± 4.1	30.3 ± 7.9	492
	0.2 µM	13.7 ± 5.2, N=860	23.9 ± 3.7	52.4 ± 10.2	23.7 ± 6.5	280
	DMSO2	19.5 ± 3.6, N=829	28.0 ± 2.5	47.7 ± 5.3	24.3 ± 2.8	218
	1 µM	8.0 ± 2.9, N=829	32.7 ± 2.9	67.3 ± 2.9	0	486
	DMSO3	11.5 ± 1.6, N=844	27.8 ± 10.2	53.1 ± 3.6	19.1 ± 10.8	400
	5 µM	1.4 ± 0.1, N=818	54.1 ± 16.0	45.9 ± 16.0	0	194
CHKi	DMSO	9.5 ± 1.5, N=563	27.4 ± 5.7	53.4 ± 7.4	18.8 ± 1.7	189
	1 µM PF-477736	2.6 ± 0.1, N=386	38.3 ± 2.4	61.7 ± 2.4	0	192
	1 µM LY2603618	4.4 ± 2.0, N=397	38.3 ± 7.0	61.8 ± 7.0	0	133

Mean, standard deviation and total number of cells counted (N) are shown.

Supplementary table 4. Average number of recombination foci in cultured spermatocytes at the indicated stages of prophase.

		Early leptonema	Late leptonema	Early zygonema	Late zygonema
RAD51	D14 – untreated	157.7 ± 25.0, N=6	137.8 ± 36.1, N=21	111.2 ± 24.8, N=39	63.0 ± 26.8, N=24
	DMSO3-AZ20 treatment	121.7 ± 32.0, N=7	141.6 ± 37.0, N=16	112.7 ± 34.2, N=27	61.5 ± 17.3, N=28
	5 µM AZ20	21.8 ± 21.3, N=16	16.6 ± 16.6, N=14	13.9 ± 14.6, N=42	8.9 ± 11.0, N=21
	DMSO-CHK1i treatment	82.2 ± 49.9, N=25	126.4 ± 46.7, N=37	136.9 ± 39.8, N=19	94.6 ± 32.3, N=21
	1 µM PF-477736	23.5 ± 18.5, N=17	41.4 ± 22.7, N=37	43.9 ± 29.6, N=24	31.5 ± 17.9, N=22
	1 µM LY2603618	23.6 ± 16.2, N=22	46.2 ± 39.8, N=28	54.2 ± 35.2, N=24	48.2 ± 19.9, N=12
RPA	D14 – untreated	152.3 ± 18.46, N=13	150.6 ± 24.7, N=11	147.4 ± 44.2, N=16	130.5 ± 31.5, N=12
	DMSO3-AZ20 treatment	154.0 ± 33.2, N=13	114.3 ± 21.4, N=12	136.6 ± 35.1, N=16	96.76 ± 30.9, N=17
	5 µM AZ20	200.2 ± 80.6, N=17	149.9 ± 33.9, N=11	133.7 ± 58.2, N=12	81.4 ± 11.6, N=5

Mean, standard deviation and total number of cells counted (N) are shown.
